# Strain-limited biofilm regulation through the Brg1-Rme1 circuit in *Candida albicans*

**DOI:** 10.1128/msphere.00980-24

**Published:** 2024-12-31

**Authors:** Min-Ju Kim, Aaron P. Mitchell

**Affiliations:** 1 Department of Microbiology, University of Georgia, Athens, Georgia, USA; University of Guelph, Guelph, Ontario, Canada

**Keywords:** *Candida albicans*, strain variation, hypha formation, biofilm

## Abstract

Prominent virulence traits of *Candida albicans* include its ability to produce filamentous hyphal cells and grow as a biofilm. These traits are under control of numerous transcription factors (TFs), including Brg1 and Rme1. In the reference strain SC5314, a *brg1*Δ/Δ mutant has reduced levels of biofilm/filament production; a *brg1*Δ/Δ *rme1*Δ/Δ double mutant has wild-type levels of biofilm/filament production. Here, we asked whether this suppression relationship is preserved in four additional strain backgrounds: P76067, P57055, P87, and P75010. These strains represent diverse clades and biofilm/filament production abilities. We find that a *rme1*Δ/Δ mutation restores biofilm/filament production in a *brg1*Δ/Δ mutant of P76067, but not in *brg1*Δ/Δ mutants of P57055, P87, and P75010. We speculate that variation in activities of two functionally related TFs, Nrg1, and Ume6, may cause the strain-limited impact of the *rme1*Δ/Δ mutation.

**IMPORTANCE:**

*Candida albicans* is a widespread fungal pathogen. The regulatory circuitry underlying virulence traits is well studied in the reference strain background, but not in other clinical isolate backgrounds. Here, we describe a pronounced example of strain variation in the control of two prominent virulence traits, biofilm formation and filamentation.

## OBSERVATION

*Candida albicans* is a prominent fungal commensal and pathogen of humans (1). One key virulence trait is its ability to make filamentous hyphae, which are required for pathogenicity in most animal infection models (1–3), and for biofilm formation, the growth form that is the basis for device-associated infection (4, 5). Hypha formation depends upon a group of signaling pathways (2) that form an interconnected network (6, 7). The downstream output that is governed by many of these regulators is a set of genes, called hypha-associated genes, whose RNA levels are much higher in hyphal cells than in yeast cells (2). Hypha-associated genes are direct targets of some of the transcription factors (TFs) (those TFs bind to the target gene promoter/regulatory region) and indirect targets of others (those TFs bind elsewhere in the genome). The products of hypha-associated genes include adhesins, secreted hydrolases, Candidalysin toxin, and determinants of cell polarity, hyphal growth, and biofilm development (7–9).

Our understanding of hyphal regulation comes from detailed analysis mainly in reference strain SC5314 and auxotrophic derivatives. The use of a reference strain background has been essential to enable direct comparison of findings among research groups. However, *C. albicans* isolates vary in both genotype (10–12) and phenotype [(11–18) (reviewed in references (19, 20)], and engineered mutations may cause different phenotypes in different *C. albicans* strains (14–16, 18, 21–24), as is the case in other organisms (25–28). For genes that govern virulence traits, mutations that cause a uniformly severe defect in diverse strains can help define targets that are most promising for the development of therapeutics.

We focus here on the TFs Brg1 and Rme1. Brg1, a biofilm master regulator (6, 7), is a GATA-type TF required for expression of hypha-associated genes (29, 30). Brg1 controls most hypha-associated genes indirectly; it binds to promoter/regulatory regions of other hyphal regulatory genes (30). Rme1, a C_2_H_2_ zinc finger TF, was discovered to be a positive regulator of chlamydospore production in *C. albicans* (31). Rme1 is also a negative regulator of biofilm production, filamentation, and hypha-associated gene expression (32). Our data and prior studies argue that Rme1 functions downstream of Brg1, and that Brg1 represses *RME1* expression.

The studies of Brg1 and Rme1 summarized above were conducted primarily in the SC5314 strain background. Here, we sought to test the generality of those findings through biological assays of *brg1*Δ/Δ and *rme1*Δ/Δ mutants in four additional clinical isolate backgrounds. We chose four clinical isolates we used in previous studies of strain variation (14–16, 21, 23). The strains, first characterized in the Soll (13) and Bennett and Cuomo labs (11), include P76067 (clade 2), P57055 (clade 3), P87 (clade 4), and P75010 (clade 11). In the studies presented here, we included the reference strain SC5314 (clade 1) as well as an internal control that could be compared to our previous data (32).

**Fig 1:**
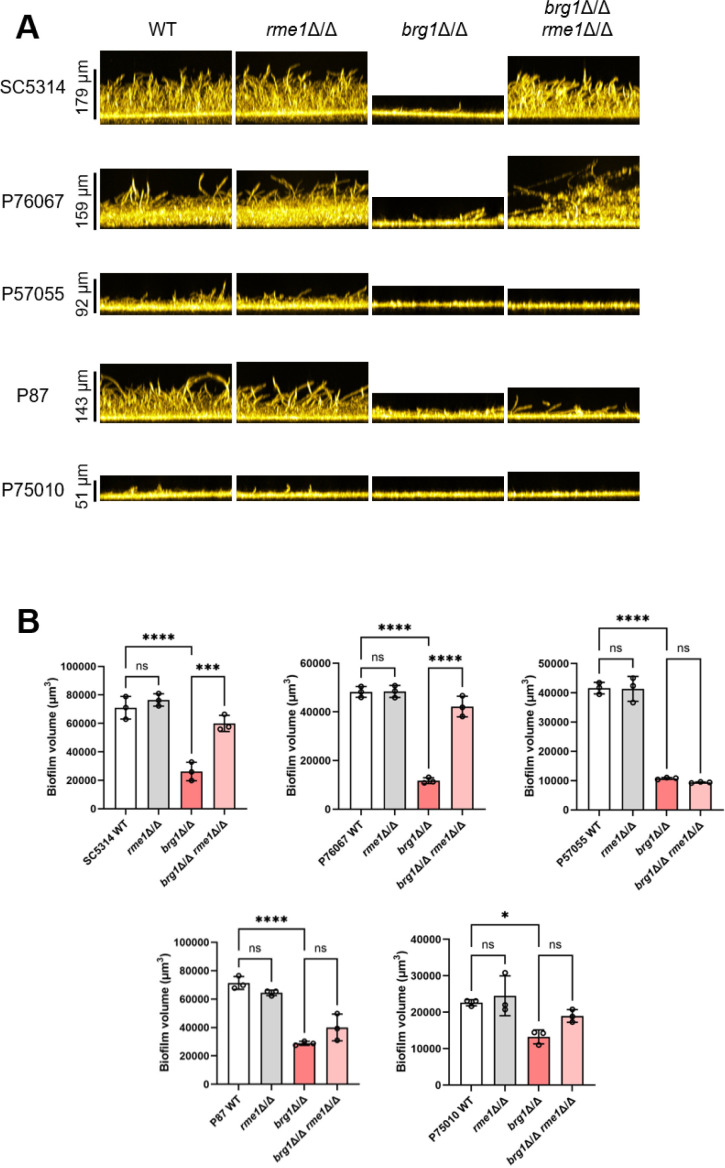
Variation in biofilm formation by *brg1*Δ/Δ *rme1*Δ/Δ double mutants as shown in side projections and volume measurements. Wild-type (WT) controls along with *rme1*Δ/Δ, *brg1*Δ/Δ, and *brg1*Δ/Δ *rme1*Δ/Δ mutants in five strain backgrounds were tested for biofilm formation under *in vitro* conditions. Strains were grown in RPMI-1640 at 37°C for 24 h in 96-well plates. Fixed biofilms were stained using calcofluor white and then imaged on a Keyence BZ-X800E fluorescence microscope. (**A**) Representative side projection views of biofilms of WT and each mutant strain are shown; scale bar lengths are indicated. (**B**) Biofilm volumes are presented. Statistical analysis was conducted using one-way ANOVA; ns indicates no significance and asterisks denote statistically significant differences. *P value < 0.05, ***P value < 0.001, and ****P value < 0.0001.

We constructed *brg1*Δ/Δ and *rme1*Δ/Δ single and double mutants in the clinical isolates using strategies described previously (Text S1). Wild-type (WT) and mutant strains were tested for biofilm formation under fairly strong inducing conditions (RPMI-1640 medium, 37°C, 24 h) in a 96-well plate format. These conditions were slightly different from our initial studies, which used RPMI-1640 + FBS in 12-well plates holding silicone squares (14). Under these conditions, WT SC5314, P76067, and P87 presented strong biofilm formation ability; P57055 presented intermediate biofilm formation ability; and P75010 presented weak biofilm formation ability (Fig. 1). The relative biofilm formation abilities observed here are largely consistent with our previous results (14–16). Importantly, the strains present a range of biofilm formation abilities.

We then tested mutant strain phenotypes. Compared to respective wild types, a *rme1*Δ/Δ mutation alone had no effect on biofilm formation, while a *brg1*Δ/Δ mutation caused a significant reduction in biofilm formation (Fig. 1). In SC5314 and P76067, the *brg1*Δ/Δ *rme1*Δ/Δ double mutants had significantly increased biofilm production compared to the *brg1*Δ/Δ single mutants. Surprisingly, though, in the P87, P57055, and P75010 backgrounds, the *brg1*Δ/Δ *rme1*Δ/Δ double mutants and *brg1*Δ/Δ single mutants had similar biofilm defects (Fig. 1). These results indicate that the impact of Rme1 on biofilm formation is strain-limited: a *rme1*Δ/Δ mutation can reverse a *brg1*Δ/Δ biofilm defect in SC5314 and P76067, but not in P87, P57055, and P75010.

We tested filamentation of the strains as well. We found previously that the *rme1*Δ/Δ mutation had little impact under planktonic growth conditions (32), and extended that result to the four additional backgrounds (Fig. S1). Under biofilm-like conditions (RPMI-1640 medium, 30 h, 37°C, sealed tubes, incubated statically), WT SC5314 and P76067 filamented efficiently, whereas the other three WT strains filamented weakly (Fig. S2) to yield hyphae at low frequency. In all strains, the *rme1*Δ/Δ mutation alone had no effect on filamentation (Fig. S2). In SC5314 and P76067, the *brg1*Δ/Δ single mutants had prominent filamentation defects, and the *brg1*Δ/Δ *rme1*Δ/Δ double mutants had significantly increased filamentation compared to the *brg1*Δ/Δ single mutants (Fig. S2). In P57055, P87, and P75010, the *brg1*Δ/Δ mutants had subtle though detectable filamentation defects, and *brg1*Δ/Δ *rme1*Δ/Δ double mutants showed similar filamentation defects compared to *brg1*Δ/Δ mutants (Fig. S2). Therefore, the impact of *RME1* on filamentation and biofilm formation is strain-limited.

**Fig 2:**
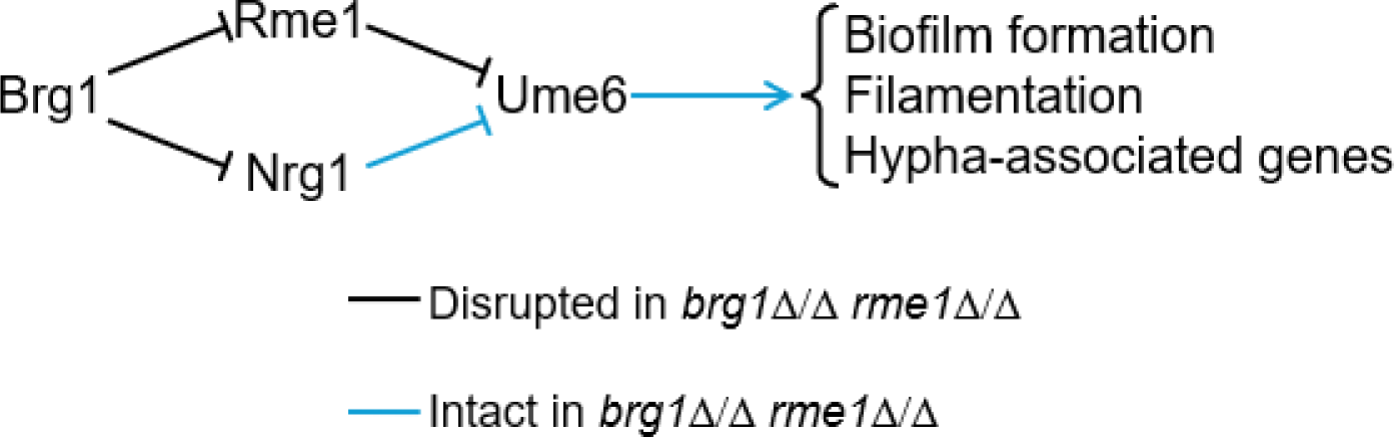
Model for functional relationships surrounding Rme1. This diagram presents a summary of relationships among the four TFs—Brg1, Nrg1, Ume6, and Rme1—with the goal of envisioning general hypotheses to explain the strain-limited impact of *rme1*Δ/Δ mutations on biofilm formation and filamentation. Brg1 and Ume6 are positive regulators of the target processes biofilm formation, filamentation, and hypha-associated gene expression; Rme1 and Nrg1 are negative regulators of the target processes. Brg1 controls the target processes indirectly through repression of *RME1* and *NRG1*; Rme1 and Nrg1 act at least in part through repression of *UME6*. The precise mechanism and direct targets of Ume6 action are not known, but it is generally accepted to act downstream of Brg1 and other biofilm and filamentation regulators. Consider the situation in *brg1*Δ/Δ *rme1*Δ/Δ double mutants, the genotype in which we detect phenotypic variation. Among the four regulators depicted, only Nrg1 and Ume6 remain. Strain differences in biofilm formation, filamentation, or hypha-associated gene expression in *brg1*Δ/Δ *rme1*Δ/Δ double mutants may result from strain differences in ability of Nrg1 to repress *UME6*, or in ability of Ume6 to activate its downstream targets. For example, under our growth conditions, SC5314 and P76067 may have low levels of Nrg1 activity, while P57055, P87, and P75010 have high levels of Nrg1 activity. See the Discussion for published studies that document Nrg1 or Ume6 activity variation.

We consider Rme1 in the context of other known Brg1-regulated TFs to try to imagine the basis for the strain-limited impact of *rme1*Δ/Δ mutations. Figure 2 depicts the functional relationships among four TFs that govern the target processes of biofilm formation, filamentation, and hypha-associated gene expression: Brg1, Nrg1, Ume6, and Rme1 (2, 3, 7, 33). Brg1 and Ume6 promote the target processes; Rme1 and Nrg1 inhibit the target processes. Brg1 acts as a repressor of *RME1* and *NRG1*; Rme1 and Nrg1 act as repressors of *UME6*. Although the Ume6 mechanism and direct targets are not known, current evidence indicates that it act downstream of other biofilm and filamentation regulators (2, 3, 33, 34).

Let us consider *brg1*Δ/Δ *rme1*Δ/Δ double mutants, whose phenotype is variable among strains. Of the four TFs in Fig. 2, Brg1 and Rme1 have been eliminated genetically in those strains, hence only Nrg1 and Ume6 remain. In those double mutants, strain differences in target process efficiency may reflect strain differences in (i) repression by Nrg1 of *UME6*, or (ii) activation of the target processes by Ume6. One specific hypothesis for example is that, under our growth conditions, SC5314 and P76067 may have low levels of Nrg1 activity, while P57055, P87, and P75010 have high levels of Nrg1 activity. Alternatively, SC5314 and P76067 may have high levels of Ume6 activity, while P57055, P87, and P75010 have low levels of Ume6 activity. These two hypotheses are not mutually exclusive, and some combination may exist. We note that differences in activity of Nrg1 or Ume6 may result from differences in the *NRG1* or *UME6* genes themselves, but may also result from differences in interacting TFs or the well-described pathways that control them translationally and post-translationally (33).

Several studies have shown that both Nrg1 and Ume6 activities vary among *C. albicans* isolates. In the case of Nrg1, aneuploidy of chromosome 7 alters *NRG1* gene dosage and thus impacts filamentation ability (35); chromosome 7 aneuploidy has been observed in many strains and contexts (11, 36, 37). In addition, Nrg1 output is variable among strains: while an *nrg1*Δ/Δ mutation causes increased filamentation in almost all strains under non-inducing conditions, it can cause reduced filamentation in some strains under inducing conditions (18, 22). In the case of Ume6, we note that the biofilm and gene expression defects of *ume6*Δ/Δ mutants are quite variable among strains (14). These observations imply that Ume6-compensatory activities are variable among strains, and hence, the impact of Ume6-dependent activation of target processes may vary. In fact, *UME6* expression levels are more variable among *C. albicans* strains than many other biofilm and filamentation regulators (16, 22). Both of these TFs are known to influence colonization ability and host interaction (22, 38, 39), so selective forces associated with commensalism may drive variation in Nrg1 and Ume6 outputs.

## SUPPLEMENTAL MATERIAL

Supplemental material msphere.00980-24-s0001.pdf Text S1, Figures S1 and S2, and Table S1.

ASM does not own the copyrights to Supplemental Material that may be linked to, or accessed through, an article. The authors have granted ASM a non-exclusive, world-wide license to publish the Supplemental Material files. Please contact the corresponding author directly for reuse.

## References

[1] Katsipoulaki M, Stappers MHT, Malavia-Jones D, Brunke S, Hube B, Gow NAR. 2024. Candida albicans and Candida glabrata: global priority pathogens. Microbiol Mol Biol Rev 88:e0002123. doi:10.1128/mmbr.00021-23 38832801 PMC11332356

[2] Basso V, d’Enfert C, Znaidi S, Bachellier-Bassi S. 2019. From genes to networks: the regulatory circuitry controlling Candida albicans morphogenesis. Curr Top Microbiol Immunol 422:61–99. doi:10.1007/82_2018_144 30368597

[3] Noble SM, Gianetti BA, Witchley JN. 2017. Candida albicans cell-type switching and functional plasticity in the mammalian host. Nat Rev Microbiol 15:96–108. doi:10.1038/nrmicro.2016.157 27867199 PMC5957277

[4] Soriano A, Honore PM, Puerta-Alcalde P, Garcia-Vidal C, Pagotto A, Gonçalves-Bradley DC, Verweij PE. 2023. Invasive candidiasis: current clinical challenges and unmet needs in adult populations. J Antimicrob Chemother 78:1569–1585. doi:10.1093/jac/dkad139 37220664 PMC10320127

[5] Desai JV, Mitchell AP, Andes DR. 2014. Fungal biofilms, drug resistance, and recurrent infection. Cold Spring Harb Perspect Med 4:a019729. doi:10.1101/cshperspect.a019729 25274758 PMC4200207

[6] Rodriguez DL, Quail MM, Hernday AD, Nobile CJ. 2020. Transcriptional circuits regulating developmental processes in Candida albicans. Front Cell Infect Microbiol 10:605711. doi:10.3389/fcimb.2020.605711 33425784 PMC7793994

[7] Lohse MB, Gulati M, Johnson AD, Nobile CJ. 2018. Development and regulation of single- and multi-species Candida albicans biofilms. Nat Rev Microbiol 16:19–31. doi:10.1038/nrmicro.2017.107 29062072 PMC5726514

[8] Wilson D, Naglik JR, Hube B. 2016. The missing link between Candida albicans hyphal morphogenesis and host cell damage. PLoS Pathog 12:e1005867. doi:10.1371/journal.ppat.1005867 27764260 PMC5072684

[9] Mayer FL, Wilson D, Hube B. 2013. Candida albicans pathogenicity mechanisms. Virulence 4:119–128. doi:10.4161/viru.22913 23302789 PMC3654610

[10] Hamlin JAP, Dias GB, Bergman CM, Bensasson D. 2019. Phased diploid genome assemblies for three strains of Candida albicans from oak trees. G3 (Bethesda) 9:3547–3554. doi:10.1534/g3.119.400486 31540974 PMC6829152

[11] Hirakawa MP, Martinez DA, Sakthikumar S, Anderson MZ, Berlin A, Gujja S, Zeng Q, Zisson E, Wang JM, Greenberg JM, Berman J, Bennett RJ, Cuomo CA. 2015. Genetic and phenotypic intra-species variation in Candida albicans. Genome Res 25:413–425. doi:10.1101/gr.174623.114 25504520 PMC4352881

[12] Ropars J, Maufrais C, Diogo D, Marcet-Houben M, Perin A, Sertour N, Mosca K, Permal E, Laval G, Bouchier C, et al.. 2018. Gene flow contributes to diversification of the major fungal pathogen Candida albicans. Nat Commun 9:2253. doi:10.1038/s41467-018-04787-4 29884848 PMC5993739

[13] Wu W, Lockhart SR, Pujol C, Srikantha T, Soll DR. 2007. Heterozygosity of genes on the sex chromosome regulates Candida albicans virulence . Mol Microbiol 64:1587–1604. doi:10.1111/j.1365-2958.2007.05759.x 17555440

[14] Huang MY, Woolford CA, May G, McManus CJ, Mitchell AP. 2019. Circuit diversification in a biofilm regulatory network. PLoS Pathog 15:e1007787. doi:10.1371/journal.ppat.1007787 31116789 PMC6530872

[15] Do E, Cravener MV, Huang MY, May G, McManus CJ, Mitchell AP. 2022. Collaboration between antagonistic cell type regulators governs natural variation in the Candida albicans biofilm and hyphal gene expression network. MBio 13:e0193722. doi:10.1128/mbio.01937-22 35993746 PMC9600859

[16] Cravener MV, Do E, May G, Zarnowski R, Andes DR, McManus CJ, Mitchell AP. 2023. Reinforcement amid genetic diversity in the Candida albicans biofilm regulatory network. PLoS Pathog 19:e1011109. doi:10.1371/journal.ppat.1011109 36696432 PMC9901766

[17] Glazier VE, Kramara J, Ollinger T, Solis NV, Zarnowski R, Wakade RS, Kim MJ, Weigel GJ, Liang SH, Bennett RJ, Wellington M, Andes DR, Stamnes MA, Filler SG, Krysan DJ. 2023. The Candida albicans reference strain SC5314 contains a rare, dominant allele of the transcription factor Rob1 that modulates filamentation, biofilm formation, and oral commensalism. MBio 14:e0152123. doi:10.1128/mbio.01521-23 37737633 PMC10653842

[18] Mao Y, Solis NV, Filler SG, Mitchell AP. 2023. Functional dichotomy for a hyphal repressor in Candida albicans. MBio 14:e0013423. doi:10.1128/mbio.00134-23 36883818 PMC10127614

[19] Lindemann-Perez E, Perez JC. 2024. Candida albicans natural diversity: a resource to dissect fungal commensalism and pathogenesis. Curr Opin Microbiol 80:102493. doi:10.1016/j.mib.2024.102493 38833793 PMC12743322

[20] Anderson MZ, Dietz SM. 2024. Evolution and strain diversity advance exploration of Candida albicans biology. mSphere 9:e0064123. doi:10.1128/msphere.00641-23 39012122 PMC11351040

[21] Xiong L, Goerlich K, Do E, Mitchell AP. 2024. Strain variation in the Candida albicans iron limitation response. mSphere 9:e0037224. doi:10.1128/msphere.00372-24 38980069 PMC11288005

[22] Wakade RS, Wellington M, Krysan DJ. 2024. The role of the C. albicans transcriptional repressor NRG1 during filamentation and disseminated candidiasis is strain dependent. mSphere 9:e0078523. doi:10.1128/msphere.00785-23 38376205 PMC10964420

[23] Kim MJ, White AM, Mitchell AP. 2024. Strain variation in Candida albicans glycolytic gene regulation. mSphere. doi:10.1128/msphere.00579-24:e0057924 PMC11580466 39431903

[24] Sharma A, Solis NV, Huang MY, Lanni F, Filler SG, Mitchell AP. 2023. Hgc1 independence of biofilm hyphae in Candida albicans. MBio 14:e0349822. doi:10.1128/mbio.03498-22 36779720 PMC10128054

[25] Hartman JL, Garvik B, Hartwell L. 2001. Principles for the buffering of genetic variation. Science 291:1001–1004. doi:10.1126/science.1056072 11232561

[26] Dowell RD, Ryan O, Jansen A, Cheung D, Agarwala S, Danford T, Bernstein DA, Rolfe PA, Heisler LE, Chin B, Nislow C, Giaever G, Phillips PC, Fink GR, Gifford DK, Boone C. 2010. Genotype to phenotype: a complex problem. Science 328:469. doi:10.1126/science.1189015 20413493 PMC4412269

[27] Chandler CH, Chari S, Dworkin I. 2013. Does your gene need a background check? How genetic background impacts the analysis of mutations, genes, and evolution. Trends Genet 29:358–366. doi:10.1016/j.tig.2013.01.009 23453263 PMC3692003

[28] Chow CY. 2016. Bringing genetic background into focus. Nat Rev Genet 17:63–64. doi:10.1038/nrg.2015.9 26659016

[29] Du H, Guan G, Xie J, Sun Y, Tong Y, Zhang L, Huang G. 2012. Roles of Candida albicans Gat2, a GATA-type zinc finger transcription factor, in biofilm formation, filamentous growth and virulence. PLoS One 7:e29707. doi:10.1371/journal.pone.0029707 22276126 PMC3261855

[30] Nobile CJ, Fox EP, Nett JE, Sorrells TR, Mitrovich QM, Hernday AD, Tuch BB, Andes DR, Johnson AD. 2012. A recently evolved transcriptional network controls biofilm development in Candida albicans. Cell 148:126–138. doi:10.1016/j.cell.2011.10.048 22265407 PMC3266547

[31] Hernández-Cervantes A, Znaidi S, van Wijlick L, Denega I, Basso V, Ropars J, Sertour N, Sullivan D, Moran G, Basmaciyan L, Bon F, Dalle F, Bougnoux M-E, Boekhout T, Yang Y, Li Z, Bachellier-Bassi S, d’Enfert C. 2020. A conserved regulator controls asexual sporulation in the fungal pathogen Candida albicans. Nat Commun 11:6224. doi:10.1038/s41467-020-20010-9 33277479 PMC7718266

[32] Kim MJ, Cravener M, Solis N, Filler SG, Mitchell AP. 2024. A Brg1-Rme1 circuit in Candida albicans hyphal gene regulation. MBio 15:e0187224. doi:10.1128/mbio.01872-24 39078139 PMC11389389

[33] Lu Y, Su C, Liu H. 2014. Candida albicans hyphal initiation and elongation. Trends Microbiol 22:707–714. doi:10.1016/j.tim.2014.09.001 25262420 PMC4256103

[34] Banerjee M, Uppuluri P, Zhao XR, Carlisle PL, Vipulanandan G, Villar CC, López-Ribot JL, Kadosh D. 2013. Expression of UME6, a key regulator of Candida albicans hyphal development, enhances biofilm formation via Hgc1- and Sun41-dependent mechanisms. Eukaryot Cell 12:224–232. doi:10.1128/EC.00163-12 23223035 PMC3571304

[35] Kakade P, Sircaik S, Maufrais C, Ene IV, Bennett RJ. 2023. Aneuploidy and gene dosage regulate filamentation and host colonization by Candida albicans. Proc Natl Acad Sci U S A 120:e2218163120. doi:10.1073/pnas.2218163120 36893271 PMC10089209

[36] Ma Q, Ola M, Iracane E, Butler G. 2019. Susceptibility to medium-chain fatty acids is associated with trisomy of chromosome 7 in Candida albicans. mSphere 4:e00402-19. doi:10.1128/mSphere.00402-19 31243082 PMC6595153

[37] Ene IV, Farrer RA, Hirakawa MP, Agwamba K, Cuomo CA, Bennett RJ. 2018. Global analysis of mutations driving microevolution of a heterozygous diploid fungal pathogen. Proc Natl Acad Sci USA 115. doi:10.1073/pnas.1806002115 PMC6140516 30150418

[38] Shao T-Y, Kakade P, Witchley JN, Frazer C, Murray KL, Ene IV, Haslam DB, Hagan T, Noble SM, Bennett RJ, Way SS. 2022. Candida albicans oscillating UME6 expression during intestinal colonization primes systemic Th17 protective immunity. Cell Rep 39:110837. doi:10.1016/j.celrep.2022.110837 35584674 PMC9196946

[39] Witchley JN, Basso P, Brimacombe CA, Abon NV, Noble SM. 2021. Recording of DNA-binding events reveals the importance of a repurposed Candida albicans regulatory network for gut commensalism. Cell Host Microbe 29:1002–1013. doi:10.1016/j.chom.2021.03.019 33915113 PMC8216204

