# Supplementary material for: Strain-limited biofilm regulation through the Brg1-Rme1 circuit in Candida albicans
Source: mSphere. 2024 Dec 31;10(1):e00980-24. doi: 10.1128/msphere.00980-24 (PMC11774020; doi:10.1128/msphere.00980-24)
Supplement: Supplemental material — Text S1, Figures S1 and S2, and Table S1. [file msphere.00980-24-s0001.pdf]

**TEXT S1. Detailed methods**

**Strains and media.** *C. albicans* strains SC5314, P76067, P57055, P87 and P75010 and their derived *his1*Δ/Δ mutants (1) were used as transformation recipients. Strains in 15% glycerol solution were frozen at -80°C for long term storage. Before all experiments, strains were grown on YPD (2% Bacto peptone, 2% dextrose, 1% yeast extract) solid medium (2% Bacto agar) at 30°C for 48 hours and cultured overnight in YPD liquid medium in a tissue culture rotator at 30°C with agitation. For construction of *rme1*Δ/Δ mutants, transformant colonies were selected on CSM-His solid medium (1.7% Difco yeast nitrogen base with ammonium sulfate with amino acid supplement lacking histidine, 2% dextrose, 2% Bacto agar). For construction of *brg1*Δ/Δ *rme1*Δ/Δ mutants, the colonies were chosen on YPD + 400 µg/mL nourseothricin (clonNAT; Gold Biotechnology) solid medium. Liquid RPMI-1640 medium (Sigma-Aldrich, Inc., St. Louis), adjusted to pH 7.4, was used for biofilm formation and filamentation assays in both planktonic and biofilm-like conditions (2, 3). All strains used in this study and their genotypes can be found in Table S1A.

**Transformation of *C. albicans* strains.** Homozygous *rme1*Δ/Δ strains were generated in SC5314, P76067, P57055, P87, and P75010 *his1*Δ::r3*NAT1*r3 strains by integrating the C.d.*HIS1* marker at the *RME1* locus using a primer set of RME1 Del rHIS1r SapI/F – RME1 Del rHis1r KpnI/R with amplified Cas9. The transient CRISPR protocol was used (4), occasionally with modifications for marker recycling (5). SC5314 *rme1*Δ/Δ (MC347), P76067 *rme1*Δ/Δ (MC342), P57055 *rme1*Δ/Δ (MC345), P87 *rme1*Δ/Δ (MK971), and P75010 *rme1*Δ/Δ (MK978) were constructed. All oligos and plasmids used in this work can be found in Table S1B and S1C.

Homozygous *brg1*Δ/Δ *rme1*Δ/Δ strains were generated in the SC5314, P76067, P57055, P87, and P75010 *brg1*Δ/Δ strain backgrounds. The *brg1*Δ/Δ mutants from five clinical isolates were made sensitive to nourseothricin by recycling the *NAT1* marker at the *his1*Δ/Δ locus when *BRG1* was deleted (5). The *NAT1* marker was amplified from plasmid pNAT (4) with 80 bp of flanking homology from the up and downstream of the *RME1* ORF region being deleted using a primer set of RME1 Del Nat1/F – RME1 Del Nat1/R. Transformant genotypes were verified using two primer sets: RME1 check up/F – NAT1 check int/R and RME1 check up/F – RME1 check int/R. SC5314 *brg1*Δ/Δ *rme1*Δ/Δ (MK939), P76067 *brg1*Δ/Δ *rme1*Δ/Δ (MK955), P57055 *brg1*Δ/Δ *rme1*Δ/Δ (MK957), P87 *brg1*Δ/Δ *rme1*Δ/Δ (MK958), P75010 *brg1*Δ/Δ *rme1*Δ/Δ (MK961) were constructed.

**Biofilm formation assays.** Biofilm formation assays were examined in a 96 well plate format (Greiner 96-well plate; Cat #. 655090) as described previously (3, 6-8). Biofilms were stained with calcofluor white, clarified with thiodiethanol and imaged on Keyence BZ-X800E fluorescence microscope using 20X with 2X digital zoom.

**Biofilm image processing.** Apical and side view projections of biofilms were observed from Z-stack images as described previously (3, 6-8). The Z-stacks were combined and processed using FIJI software program (ImageJ v1.53) (9). First, Z-stacks were converted to 32-bit from 8-bit and background signal was subtracted using the background subtract plugin. To obtain side view images, Z-stack reslicing and subsequent maximum intensity projection were conducted. Next, the side view images were rescaled based on the objective used for Keyence derived images. Apical view projections of the biofilms were created using maximum-intensity Z-projection. For both

side and apical view images, brightness was adjusted for clarity and yellow coloration was added.

**Filamentation assays in planktonic and biofilm-like conditions.** Assays were carried out as described previously (1, 3), with the modification that we used RPMI-1640 medium without FBS. Wild-type, *rme1*Δ/Δ, *brg1*Δ/Δ, *brg1*Δ/Δ *rme1*Δ/Δ, and *brg1*Δ/Δ *rme1*Δ/Δ + *RME1* strains were grown overnight in YPD liquid medium in a tissue culture rotator at 30°C. The pre-warmed 5 mL aliquots of RPMI-1640 medium were inoculated from the overnight cultures to achieve an $OD_{600}$ of 0.5, followed by incubation at 37°C for 30 hours, with agitation at 60 rpm or without agitation. Samples for filamentation were collected through centrifugation and then fixed in 4% formaldehyde in PBS solution for 15 minutes. Afterwards, the samples were washed in PBS twice and stained with calcofluor white. Stained cells were imaged with a Zeiss Axiovert 200 fluorescence microscope. Hyphal induction was determined by measuring filament unit length either from yeast cell to filament tip or between septations in ImageJ. At least 100 filament units were quantified for each strain from four separate fields of view.

**Figure S1. Filamentation assays of *brg1*Δ/Δ *rme1*Δ/Δ mutants in five strain backgrounds under planktonic conditions.** Wild-type (WT), *rme1*Δ/Δ, *brg1*Δ/Δ, and *brg1*Δ/Δ *rme1*Δ/Δ strains of each background were grown in RPMI-1640 medium under planktonic conditions (RPMI-1640 medium, 30 hours, 37°C, with vigorous shaking). **(A)** Representative images are shown. **(B)** Cell length distributions are shown. The white scale bars in each image are 50 µm in length. Cell length distributions reflect measurements of at least 4 fields of view and 100 cells. Statistical analysis was conducted using a one-way ANOVA; ns indicates not significant and asterisks denote statistically significant differences. **P* value < 0.05, ***P* value < 0.01 and *****P* value < 0.0001.

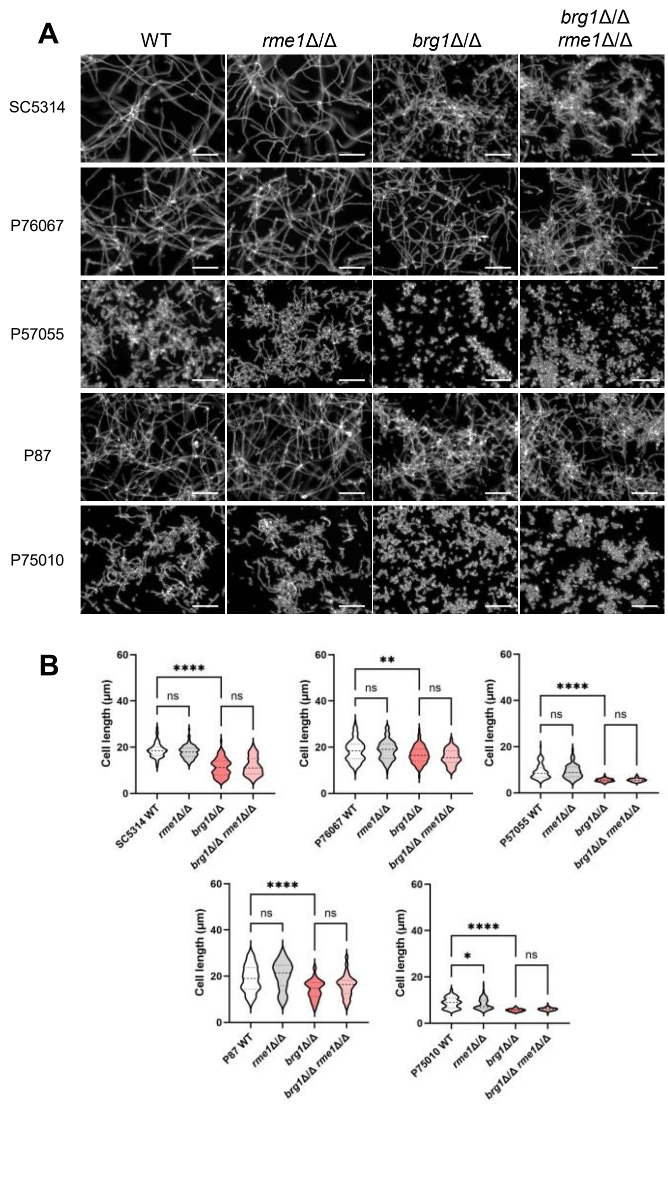

**Figure S2. Filamentation assays of *brg1*Δ/Δ *rme1*Δ/Δ mutants in five strain backgrounds under biofilm-like conditions.** Wild-type (WT), *rme1*Δ/Δ, *brg1*Δ/Δ, and *brg1*Δ/Δ *rme1*Δ/Δ strains of each background were grown in RPMI-1640 medium under biofilm-like conditions (RPMI-1640 medium, 30 hours, 37°C, sealed tubes, incubated statically). **(A)** Representative images are shown. **(B)** Cell length distributions are shown. The white scale bars in each image are 50 µm in length. Cell length distributions reflect measurements of at least 4 fields of view and 100 cells. Statistical analysis was conducted using a one-way ANOVA; ns indicates not significant and asterisks denote statistically significant differences. ***P* value < 0.01 and *****P* value < 0.0001.

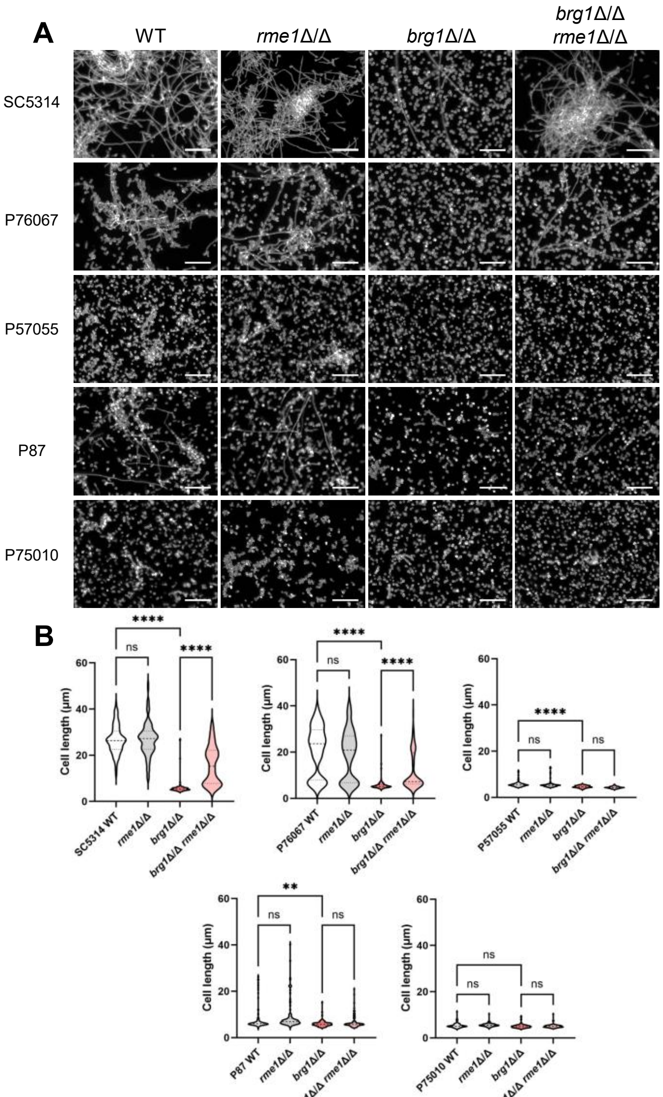

**Table S1A. Strain list**

| Strain Numbe r | Parent Strain | Markers | Description / Genotype | Notes | Reference |
|---|---|---|---|---|---|
| MC22 | N/A | | SC5314 wild-type | | (10) |
| MC99 | N/A | | Clinical isolate P76067 | | (11) |
| MC69 | N/A | | Clinical isolate P57055 | | (11) |
| MC102 | N/A | | Clinical isolate P87 | | (11) |
| MC63 | N/A | | Clinical isolate P75010 | | (11) |
| MC5 | MC22 | His+, NatR | SC5314 *his1Δ::r3NAT1r3/his1Δ::r3NAT1r3* | SC5314 *his1*Δ/Δ mutant | (7) |
| MC1 | MC99 | His+, NatR | P76067 *his1Δ::r3NAT1r3/his1Δ::r3NAT1r3* | P76067 *his1*Δ/Δ mutant | (7) |
| MC2 | MC69 | His+, NatR | P57055 *his1Δ::r3NAT1r3/his1Δ::r3NAT1r3* | P57055 *his1*Δ/Δ mutant | (7) |
| MC3 | MC102 | His+, NatR | P87 *his1Δ::r3NAT1r3/his1Δ::r3NAT1r3* | P87 *his1*Δ/Δ mutant | (7) |
| MC4 | MC63 | His+, NatR | P75010 *his1Δ::r3NAT1r3/his1Δ::r3NAT1r3* | P75010 *his1*Δ/Δ mutant | (7) |
| MC347 | MC5 | His+, NatR | *rme1Δ::r1HIS1r1/rme1Δ::r1HIS1r1, his1Δ::r3NAT1r3/his1Δ::r3NAT1r3* | SC5314 *rme1*Δ/Δ mutant | (3) |
| MC342 | MC1 | His+, NatR | *rme1Δ::r1HIS1r1/rme1Δ::r1HIS1r1, his1Δ::r3NAT1r3/his1Δ::r3NAT1r3* | P76067 *rme1*Δ/Δ mutant | This study |
| MC345 | MC2 | His+, NatR | *rme1Δ::r1HIS1r1/rme1Δ::r1HIS1r1, his1Δ::r3NAT1r3/his1Δ::r3NAT1r3* | P57055 *rme1*Δ/Δ mutant | This study |
| MK971 | MC3 | His+, NatR | *rme1Δ::r1HIS1r1/rme1Δ::r1HIS1r1, his1Δ::r3NAT1r3/his1Δ::r3NAT1r3* | P87 *rme1*Δ/Δ mutant | This study |
| MK978 | MC4 | His+, NatR | *rme1Δ::r1HIS1r1/rme1Δ::r1HIS1r1, his1Δ::r3NAT1r3/his1Δ::r3NAT1r3* | P75010 *rme1*Δ/Δ mutant | This study |
| FUN44 | MC5 | His+, NatS | *brg1Δ::r1HIS1r1/brg1Δ::r1HIS1r1, his1Δ::r3/his1Δ::r3* | SC5314 *brg1*Δ/Δ mutant | (1) |
| FUN45 | MC1 | His+, NatS | *brg1Δ::r1HIS1r1/brg1Δ::r1HIS1r1, his1Δ::r3/his1Δ::r3* | P76067 *brg1*Δ/Δ mutant | (1) |
| FUN46 | MC2 | His+, NatS | *brg1Δ::r1HIS1r1/brg1Δ::r1HIS1r1, his1Δ::r3/his1Δ::r3* | P57055 *brg1*Δ/Δ mutant | (1) |

| | | | | | |
|---|---|---|---|---|---|
| FUN47 | MC3 | His+, NatS | *brg1Δ::r1HIS1r1/brg1Δ::r1HIS1r1, his1Δ::r3/his1Δ::r3* | P87 *brg1*Δ/Δ mutant | (1) |
| FUN48 | MC4 | His+, NatS | *brg1Δ::r1HIS1r1/brg1Δ::r1HIS1r1, his1Δ::r3/his1Δ::r3* | P75010 *brg1*Δ/Δ mutant | (1) |
| MK939 | FUN44 | His+, NatR | *brg1Δ::r1HIS1r1/brg1Δ::r1HIS1r1, rme1Δ::NAT1/rme1Δ::NAT1, his1Δ::r3/his1Δ::r3* | SC5314 *brg1*Δ/Δ *rme1*Δ/Δ mutant | (3) |
| MK955 | FUN45 | His+, NatR | *brg1Δ::r1HIS1r1/brg1Δ::r1HIS1r1, rme1Δ::NAT1/rme1Δ::NAT1, his1Δ::r3/his1Δ::r3* | P76067 *brg1*Δ/Δ *rme1*Δ/Δ mutant | This study |
| MK957 | FUN46 | His+, NatR | *brg1Δ::r1HIS1r1/brg1Δ::r1HIS1r1, rme1Δ::NAT1/rme1Δ::NAT1, his1Δ::r3/his1Δ::r3* | P57055 *brg1*Δ/Δ *rme1*Δ/Δ mutant | This study |
| MK958 | FUN47 | His+, NatR | *brg1Δ::r1HIS1r1/brg1Δ::r1HIS1r1, rme1Δ::NAT1/rme1Δ::NAT1, his1Δ::r3/his1Δ::r3* | P87 *brg1*Δ/Δ *rme1*Δ/Δ mutant | This study |
| MK961 | FUN48 | His+, NatR | *brg1Δ::r1HIS1r1/brg1Δ::r1HIS1r1, rme1Δ::NAT1/rme1Δ::NAT1, his1Δ::r3/his1Δ::r3* | P75010 *brg1*Δ/Δ *rme1*Δ/Δ mutant | This study |

**Table S1B. Oligo list**

| Name | Sequence (5' → 3') |
|---|---|
| CaCas9/F | ATCTCATTAGATTTGGAACTTGTGGGTT |
| CaCas9/R | TTCGAGCGTCCCAAAACCTTCT |
| SNR52/F | AAGAAAGAAAGAAAACCAGGAGTGAA |
| sgRNA/R | ACAAATATTTAAACTCGGGACCTGG |
| SNR52/N | GCGGCCGCAAGTGATTAGACT |
| sgRNA/N | GCAGCTCAGTGATTAAGAGTAAAGATGG |
| RME1 sgRNA/F | aatctgctatcctccaatggGTTTTAGAGCTAGAAATAGCAAGTTAAA |
| RME1 SNR52/R | ccattggaggatagcagattCAAATTAAAAATAGTTTACGCAAGTC |
| RME1 Del rHIS1r SapI/F | tttgtctttggaaagcttattttctttttgttatttcagttcagttcagtctactgccccttccatttcctctcctttccCTCGAGGTCGACGGTATCG |
| RME1 Del rHIS1r Kpn1/R | aataaaaaaataaaaactttttctgttttaataccgaaaacttgattcttttcccgtataatgttggaggacgaaaggaaCCAATACGCAAACCGCC |
| RME1 Del Nat1/F | tttgtctttggaaagcttattttctttttgttatttcagttcagttcagtctactgccccttccatttcctctcctttccTTTCCCAGTCACGACGTT |
| RME1 Del Nat1/R | aataaaaaaataaaaactttttctgttttaataccgaaaacttgattcttttcccgtataatgttggaggacgaaaggaaGTGGAATTGTGAGCGGATA |
| RME1 chk up/F | CACACACTTAACCCACCAGCAC |
| RME1 chk int/R | GTTAACAGGTGAACCTAATGAGCAAGAGTC |
| NAT1 chk int/R | TCAATGGTGGATCAACTGGAACTTC |

**Table S1C. Plasmid list**

| Plasmid Name | Description | Marker | Reference |
|---|---|---|---|
| pV1093 | CaCas9/sgRNA expression vector | AmpR | (12) |
| pMH01 | pRS424 carrying *C.d.HIS1* from pSN52 at KpnI site | AmpR | (5) |
| pMH02 | pRS424 carrying *C.d.HIS1* from pSN52 at SapI site | AmpR | (5) |
| pNAT | NAT1 marker | AmpR | (4) |

**Supplementary material references**


1. Huang MY, Woolford CA, May G, McManus CJ, Mitchell AP. 2019. Circuit diversification in a biofilm regulatory network. PLoS Pathog 15:e1007787.
2. Xiong L, Pereira De Sa N, Zarnowski R, Huang MY, Mota Fernandes C, Lanni F, Andes DR, Del Poeta M, Mitchell AP. 2024. Biofilm-associated metabolism via ERG251 in Candida albicans. PLoS Pathog 20:e1012225.
3. Kim MJ, Cravener M, Solis N, Filler SG, Mitchell AP. 2024. A Brg1-Rme1 circuit in Candida albicans hyphal gene regulation. mBio 15:e0187224.
4. Min K, Ichikawa Y, Woolford CA, Mitchell AP. 2016. Candida albicans Gene Deletion with a Transient CRISPR-Cas9 System. mSphere 1.
5. Huang MY, Mitchell AP. 2017. Marker Recycling in Candida albicans through CRISPR-Cas9-Induced Marker Excision. mSphere 2.
6. Do E, Cravener MV, Huang MY, May G, McManus CJ, Mitchell AP. 2022. Collaboration between Antagonistic Cell Type Regulators Governs Natural Variation in the Candida albicans Biofilm and Hyphal Gene Expression Network. mBio doi:10.1128/mbio.01937-22:e0193722.
7. Cravener MV, Do E, May G, Zarnowski R, Andes DR, McManus CJ, Mitchell AP. 2023. Reinforcement amid genetic diversity in the Candida albicans biofilm regulatory network. PLoS Pathog 19:e1011109.
8. Sharma A, Solis NV, Huang MY, Lanni F, Filler SG, Mitchell AP. 2023. Hgc1 Independence of Biofilm Hyphae in Candida albicans. mBio 14:e0349822.
9. Schindelin J, Arganda-Carreras I, Frise E, Kaynig V, Longair M, Pietzsch T, Preibisch S, Rueden C, Saalfeld S, Schmid B, Tinevez JY, White DJ, Hartenstein V, Eliceiri K, Tomancak P, Cardona A. 2012. Fiji: an open-source platform for biological-image analysis. Nat Methods 9:676-82.
10. Fonzi WA, Irwin MY. 1993. Isogenic strain construction and gene mapping in Candida albicans. Genetics 134:717-28.
11. Hirakawa MP, Martinez DA, Sakthikumar S, Anderson MZ, Berlin A, Gujja S, Zeng Q, Zisson E, Wang JM, Greenberg JM, Berman J, Bennett RJ, Cuomo CA. 2015. Genetic and phenotypic intra-species variation in Candida albicans. Genome Res 25:413-25.
12. Vyas VK, Barrasa MI, Fink GR. 2015. A Candida albicans CRISPR system permits genetic engineering of essential genes and gene families. Sci Adv 1:e1500248.
